# Establishment of an Immunocompetent Metastasis Rat Model with Hepatocyte Cancer Stem Cells

**DOI:** 10.3390/cancers12123721

**Published:** 2020-12-11

**Authors:** Semon Wu, I-Chieh Tseng, Wen-Cheng Huang, Cheng-Wen Su, Yu-Heng Lai, Che Lin, Alan Yueh-Luen Lee, Chan-Yen Kuo, Li-Yu Su, Ming-Cheng Lee, Te-Cheng Hsu, Chun-Hsien Yu

**Affiliations:** 1 Department of Life Science, Chinese Culture University, Taipei 11114, Taiwan; zyj17@ulive.pccu.edu.tw; 2 License Biotech, Co., Ltd., Taipei 10690, Taiwan; wencheng7373@gmail.com (W.-C.H.); chengwensu@gmail.com (C.-W.S.); 3 Department of Chemistry, Chinese Culture University, Taipei 11114, Taiwan; lyh21@ulive.pccu.edu.tw; 4 Department of Electrical Engineering and Graduate Institute of Communication Engineering, National Taiwan University, Taipei 10617, Taiwan; che.lin@gmail.com; 5 National Institute of Cancer Research, National Health Research Institutes, Zhunan, Miaoli 35053, Taiwan; alanylee@nhri.edu.tw; 6 Department of Research, Taipei Tzu Chi Hospital, Buddhist Tzu Chi Medical Foundation, Taipei 23142, Taiwan; cykuo863135@gmail.com (C.-Y.K.); julia10025@gmail.com (L.-Y.S.); xd108221@tzuchi.com.tw (M.-C.L.); 7 Department of Electrical Engineering, National Tsing Hua University, Hsinchu, Taipei 30013, Taiwan; andy810436@gmail.com; 8 Department of Pediatrics, Taipei Tzu Chi Hospital, Buddhist Tzu Chi Medical Foundation, Taipei 23142, Taiwan; 9 Department of Pediatrics, School of Medicine, Tzu Chi University, Hualien 97071, Taiwan

**Keywords:** hepatocyte cancer stem cell, cell renew, drug resistance, metastasis, xenografts

## Abstract

**Simple Summary:**

Cancer stem cells (CSCs) are considered responsible for the maintenance, metastasis, and recurrence of various tumors. Here, we isolated a novel CSCs, TW-1, which can self-renew, differentiate and resistant to sorafenib. We also established novel orthotopic and metastasis models in immunocompetent rats with TW-1. These immunocompetent animal model can provide a normal physiological condition for anti-cancer drug screening. Using machine learning, we predicted HCC (AUCs > 0.9) with eight biomarkers including 6 highly expressed in TW-1/HTC and 2 well-known biomarkers from recent HCC studies. In conclusion, our results showed that TW-1 was a novel rat CSC line, and the animal model established with TW-1, is not only useful for the study of metastasis of HCC but also preclinical cancer drug screening.

**Abstract:**

Hepatocellular carcinoma (HCC) is one of the leading causes of cancer mortality. Cancer stem cells (CSCs) are responsible for the maintenance, metastasis, and relapse of various tumors. The effects of CSCs on the tumorigenesis of HCC are still not fully understood, however. We have recently established two new rat HCC cell lines HTC and TW-1, which we isolated from diethylnitrosamine-induced rat liver cancer. Results showed that TW-1 expressed the genetic markers of CSCs, including CD133, GSTP1, CD44, CD90, and EpCAM. Moreover, TW-1 showed higher tolerance to sorafenib than HTC did. In addition, tumorigenesis and metastasis were observed in nude mice and wild-type rats with TW-1 xenografts. Finally, we combined highly expressed genes in TW-1/HTC with well-known biomarkers from recent HCC studies to predict HCC-related biomarkers and able to identify HCC with AUCs > 0.9 after machine learning. These results indicated that TW-1 was a novel rat CSC line, and the mice or rat models we established with TW-1 has great potential on HCC studies in the future.

## 1. Introduction

Hepatocellular carcinoma (HCC) is one of the most common cancers and is the leading cause of cancer mortality in many countries, including Taiwan [1]. The high mortality of HCC is mainly due to its invasive and intrahepatic metastatic outcomes. The survival of HCC patients after curative resection was also sharply reduced because of high rate of recurrence [2].

With the understanding of solid cancers, cancer stem cells (CSCs) have now become a key topic of research interest. The theory hypothesized that a small number of CSCs drive the growth, repopulation, and metastasis of solid cancers [3,4,5]. Not only CSCs share similar cell surface markers including CD44, CD24, CD133, CD166, and epithelial cell adhesion molecule (EpCAM) [4,6,7,8,9,10,11], but also self-renew, differentiate into different lineages, and utilize common signaling pathways, which happened in general pluripotent stem cells [12,13,14,15]. The significant difference between these two types of stem cells are the ability to form malignant tumors when transplanted into animals [15]. In addition, CSCs-derived tumors are resistant to chemotherapeutic agents and prone to recurrence and distant metastases.

Diethylnitrosamine (DEN)-induced liver cancer was a well-established model to study the mechanisms of HCC development in rodents [16]. In this paper, we described the isolation and characterization of two cancer cell lines from a rat model of DEN-induced HCC.

## 2. Materials and Methods

### 2.1. Diethylnitrosamine (DEN)-Induced Rat Liver Cancer

Male DPPIV-deficient F344 rats were provided by Professor Gupta of the Albert Einstein College of Medicine and were intraperitoneally injected with DEN to induce liver cancer (Figure S1) as describing in previous studies [17,18]. The rats were treated with DEN for 6 weeks and sacrificed at 48 weeks. Tumors (T1-8) were isolated, measured, and xenografted into nude mice. Only T2 was successfully grown into a solid tumor after 8 weeks. Mice injected with the liver cancer cell line, HepG2, were used as control. The animals were allowed free access to food and tap water, which is ad libitum, during the experimental procedures. All animals were husbandry according to animal ethic, and the protocols were approved by the Taipei Tzu-Chi Hospital Animal Ethics Committee (107-IACUC-028).

### 2.2. Cell Culture

T2 tumor was isolated, dissected and digested with 0.005% collagenase at 37 °C for one hour. The cell suspensions were diluted and cultured in 10 cm dish at 37 °C incubator containing 5% CO_2_ for overnight without antibiotics. Cells then incubated for another 7 days for waiting to from colonies. By using the different degree of adhesion, we isolated two homogenous cell line HTC and TW-1. HTC and TW-1 cells were maintained in a modified DMEM/F12 medium (Invitrogen, Carlsbad, CA, USA) supplemented with 10% fetal bovine serum (HyClone Laboratories, Logan, UT, USA), 80 units/mL penicillin, 80 µg/mL streptomycin, and 0.0175 mg/mL L-proline (Sigma, St. Louis, MI, USA).

### 2.3. RNA Extraction and Microarray Assay

Total RNA was extracted using TRIzol (Invitrogen, NY, USA) and RNeasy mini kits (QIAGEN, Germantown, MD, USA). Whole-genome gene expression analysis was performed on an Affymetrix Rat Genome 2.0 ST GeneChip platform according to the manufacturer’s protocol (Affymetrix, Santa Clara, CA, USA). The hybridization intensity was processed using the GeneChip Operating software (Affymetrix), and the genes were filtered based on the Affymetrix P/A/M flags to retain all samples under at least one of the experimental conditions. Grid alignment and data extraction of the images were performed by using the Transcriptome Analysis Console (TAC) software (Affymetrix). RNAs in HTC and TW-1 cells were selected after normalization using a multiple differential screening method.

### 2.4. Western Blotting

An equal amount of proteins was loaded and separated by 10% SDS-polyacrylamide gel and transferred to polyvinylidene difluoride (PVDF) membranes. The membranes were incubated with the primary and secondary antibodies for 1 h, respectively. The immunoreactive proteins were detected by using the enhanced ECL chemiluminescence Western Blotting Detection System (ChemiDoc XRS, Bio-Rad, Hercules, CA, USA). Signal strengths were quantified by using densitometric program (Image Lab, Bio-Rad, CA, USA). The original western blot pictures and all primary antibodies were shown in Figure S2.

### 2.5. Immunofluorescence Staining

The TW-1 cells were seeded in a glass cover slide with 1% gelatine for immunofluorescence staining. The sections were fixed with 4% paraformaldehyde and then incubated with the primary antibodies against CD133 (GeneTex, Irvine, CA, USA) for 2 h at 37 °C (5 μg/mL). The sections were counterstained with DAPI and mounted in Mounting Medium H-1000 (Vector Laboratories, Burlingame, CA, USA) as previously described [19]. There was no non-specific staining when the primary antibody was omitted. Fluorescent microscopic images were captured using a Nikon Eclipse 80i microscope (Nikon Optical, Tokyo, Japan).

### 2.6. Chemotherapy Resistance Assay

Five thousand cells were trypsinized and seeded onto each well of 96-well plates (Corning, Corning, NY, USA) and grew in a 200 µL culture medium. All cells were treated with 0 to 40 µM sorafenib (Sigma, St. Louis, MI, USA) for 24 h. Relative cell numbers were determined by CellTiter-Glo Luminescent Cell Viability Assay (Promega, Madison, WI, USA). Dose-response experiments were performed in triplicate. The percentage of cell survival was expressed relative to the untreated control.

### 2.7. Xenograft in Nude Mice Model and the Orthotopic and Metastasis Immunocompetent Rat Model

Male athymic nude mice (BALB/cAnN.Cg-Foxn1nu/CrlNarl) and F344 rats were purchased from the National Laboratory Animal Center in Taiwan, and inoculated subcutaneously 5 × 10^5^ HCC cells within right flanks of nude mice, or 2 × 10^6^ HCC cells in liver of F344 rats. Cells with different origin were suspended in 150 μL of DMEM/F12 (Invitrogen, Carlsbad, CA, USA) modified medium with an equal part of Matrigel (BD Biosciences, San Jose, CA, USA) before innoculated. The animals were sacrificed after one month. Tumor length (l) and width (w) were measured using an external caliper once a week. Tumor volume (v, mm^3^) was calculated using the formula v = l w^2^/2. For the orthotopic and metastasis immunocompetent model, male F344 rats were received two times of intraperitoneal injection with retrorsine (30 mg/kg) in two weeks. Then mice were implanted TW-1 cells after one week for orthotopic or two weeks for metastasis, respectively. For the control model, it was treated for retrosine only and did not inoculated any cells before sacrifice.

### 2.8. Microarray Datasets

The open-access microarray data were downloaded from the National Center for Biotechnology Information (NCBI) Gene Expression Omnibus (GEO) database (http://www.ncbi.nlm.nih.gov/geo). Five datasets, including GSE14520 (n = 488), GSE60502 (n = 36), GSE62232 (n = 91), GSE6764 (n = 75), and GSE76297 (n = 304) were utilized in our experiment. We combined these datasets with those with accession numbers 14520, 60502, 62232, and 6764 into a cohort to train the machine learning classifiers. In total, we used 552 samples for training and 138 samples for testing. The raw CEL data were pre-processed by the multi-array average (RMA) algorithm, and gene expression values were log2-transformed. The GSE76297 dataset was used for independent validation, and its series matrix with RMA-transformed gene expression profiles was available. Among the 381 HCC and 309 non-tumor samples included in the cohort, 121 HCC and 183 non-tumor samples were in the GSE76297.

### 2.9. Biomarker Selection with Single Biomarker AUC Scores

We initially constructed a list of HCC biomarkers from our experiments and related literature, including IL33, MAL2, TSPAN8, CD55, COX2, AFP, ENO1, and RBBP5. We then expanded the list by adding the top ten genes that showed at least 10 folds differences in expression between the microarray data of TW-1 and HTC (ND2, AKR1B8, S100A11, RPS29, RPL10, ND4, ND5, S100A10, GSTA1, and COX3) together with the well-known CSC biomarkers (CD133, CD90, CD326, CD13, and CD44). We ranked these biomarkers based on their single AUC scores, which we calculated using microarray data under the assumption that the predicted probabilities of the classifiers and their AUC scores were calculated. A biomarker with a high single AUC score indicated a close match to the sample label (HCC vs. non-tumor) and was retained to train the machine learning classifiers. In the end, IL33, TSPAN8, RBPP5, ENO1, CD55, CD44, S100A10, and GSTA1 were selected with singular AUCs > 0.7.

### 2.10. Experimental Details for Machine Learning Classifiers

Three types of machine learning classifiers, logistic regression (LR), support vector machine (SVM), and random forest (RF), were utilized to evaluate the discriminatory power of the selected biomarkers with high single AUC scores. To distinguish HCC from non-tumor samples, we used expression levels of the selected biomarkers as inputs to train these classifiers. All models were implemented using the scikit-learn packages in Python. We trained the LR classifier with L2 regularization and the regularization constant C = 0.1. We applied a balanced class weight on the loss function to combat class imbalance. The SVM classifier was trained with a radial basis function as its kernel, as well as a balanced class weight on the loss function. As to the RF classifier, we restricted the maximum depth of trees at five and used 1000 trees as an ensemble. All classifiers were trained and tested on the cohort first. The dataset GSE76297 was used to validate and examine the generalization abilities of the three classifiers independently.

### 2.11. Statistical Analyses

Statistical analysis was carried out with SPSS version 12.0 (SPSS Inc., Chicago, IL, USA). Clinical characteristics of the continuous variables were expressed as means ± SD and tested by student t-test or ANOVA. A value of *p* < 0.05 was considered statistically significant. The experiments were repeated at least three times independently. The receiver operating characteristic (ROC) curve was created to show the discriminatory power of a binomial classifier and the true-positive against the false-positive rate at various thresholds. The area under the ROC curve (AUC) was subsequently calculated.

## 3. Results

### 3.1. Isolation and Culture of Novel Liver Cancer Stem Cell Lines

**Figure 1:**
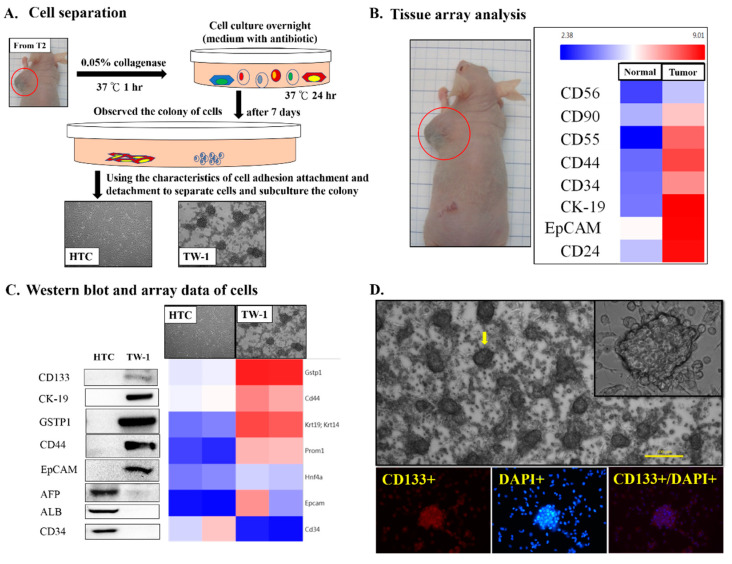
Establishment of the HTC and TW-1 cell lines. (**A**) Schematic design of the isolation of two different cell lines from T2-xenografted nude mouse. (**B**) Microarray analysis of T2-derived tumor tissue sample. The normal sample was from the adjacent non-malignant tissue. (**C**) Western blotting and microarray analyses of the HTC and TW-1 cells. (**D**) Cell morphology and immunofluorescence staining of the TW-1 cells.

**Figure 2:**
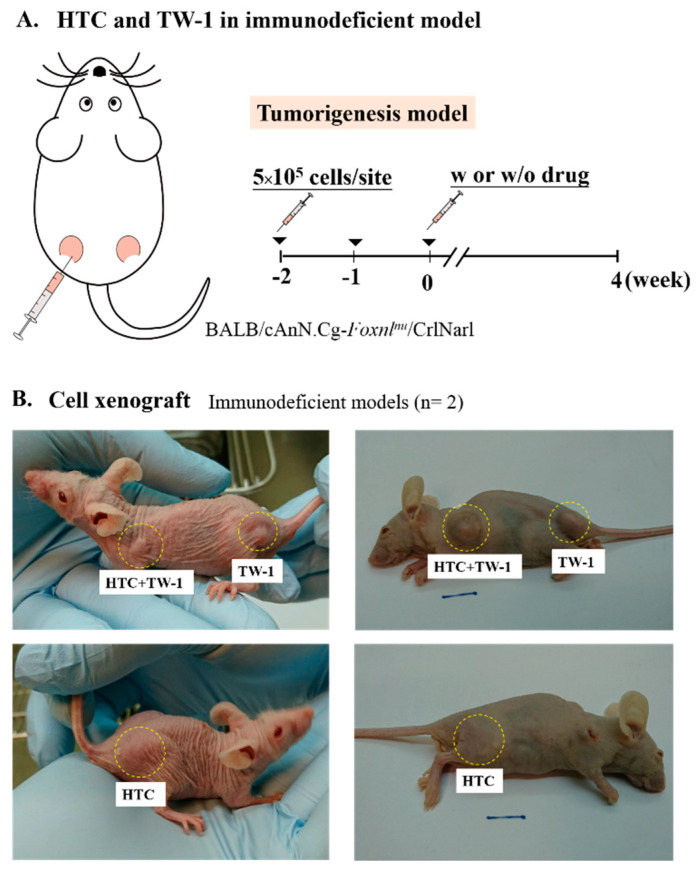
HTC and TW-1 transplantation in null mice. (**A**) The schematic design of the mouse model for HTC and TW-1 xenografts. Male immunodeficient BALB/cAnN.Cg-Foxnlnu/CrlNarl athymic nude mice were injected with HTC, TW-1 or HTC + TW-1 cells, and treated with or without sorafenib. (**B**) The appearances of xenografts in the transplanted mice.

To generate an animal model of liver cancer, DEN was intermittently administrated into rat liver to simulate the occurrence and development of human liver cancer (Figure S1). Eight solid tumors were isolated and independently xenografted into nude mice. Only tumor number 2 (T2) grew into solid cancer (Figure S1), which was suggested malignant. We isolated the solid tumor and performed microarray analysis and primary cell culture (Figure 1A). The solid tumor overexpressed various CSC-associated genes, including CD44, CD34, CD24, and EpCAM (Figure 1B). We then derived two cell lines HTC and TW-1 from the solid tumor (Figure 1A). By using Western blotting and immunofluorescence staining, TW-1 showed characteristics of CSCs, including overexpression of CD133, CK19, GSTP1, CD44, and EpCAM (Figure 1C,D). We xenografted HTC and TW-1 into nude mice to test their tumorigenicity (Figure 2A). Tumorigenesis was only observed in TW-1-xenografted mice, which indicated that TW-1 might be a novel hepatocarcinoma (HCC) line with CSC properties (Figure 2B). However, it did not express alpha-fetoprotein (AFP), one of the biomarkers of HCC.

### 3.2. TW-1 Showed the Ability to Differentiate

Previous studies suggested that about a quarter of the cancer cells may be characterized as CSCs with general stem cell properties, which showed ability to self-renewal and differentiation [20]. After culturing TW-1 for a long-term, although we did not validate the cell type that CSCs differentiated into, we did observe their ability to self-renew and differentiate into diverse cell lineages (Figure S3).

### 3.3. TW-1 Shows a High Chemotherapy Resistance In Vitro and In Vivo

**Figure 3:**
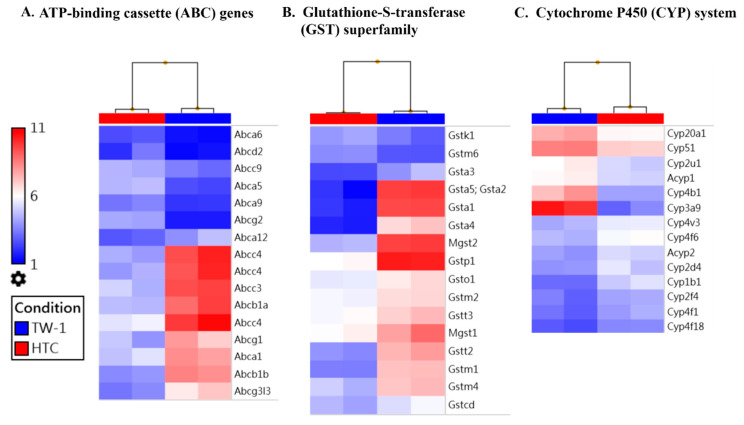
Expression of drug resistance gene families in HTC and TW-1 cells. The drug resistance genes overexpressed in the TW-1 cells included (**A**) ATP-binding cassette (ABC), (**B**) glutathione-S-transferase (GST) superfamily, and (**C**) cytochrome P450 (CYP) system.

**Figure 4:**
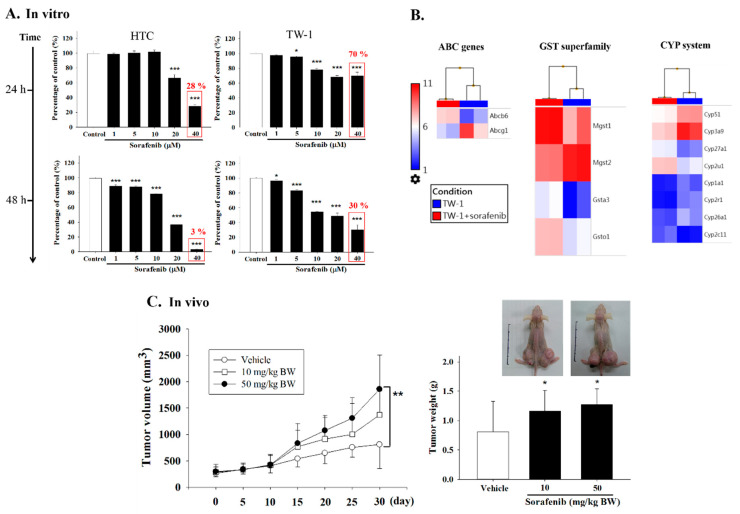
Sorafenib-induced drug resistance in HTC and TW-1 cells. (**A**) HTC and TW-1 cell lines were treated with different concentrations of sorafenib for 24 and 48 h. MTT assay was used to quantify cell viability.( *: p<0.05; ***: p<0.001, *v.s.* control.) (**B**) Microarray data showed the genes induced by sorafenib. (**C**) Null mice were treated with vehicle control or different concentrations of sorafenib two weeks after the TW-1 transplantation (the scheme was shown in Figure 3). Tumor volume and weight were measured. (*: p<0.05; **: p<0.01, *v.s.* vehicle.)

Generally, CSCs associate with the heterogeneity of tumorigenesis and systematic metastasis that cause the failure of clinical treatments [20]. Our microarray data showed profound differences between TW-1 and HTC: TW-1 had higher expression of several drug resistance related gene families, including the ATP-binding cassette (ABC), the cytochrome P450 (CYP) system, and the glutathione-S-transferase (GST) superfamily (Figure 3). We hypothesized that the differences in gene expression profiles could result in contrasting carcinogenic capacity. Therefore, we evaluated the ability of drug-resistance of HTC and TW-1 by using Luminescent Cell Viability assay after sorafenib treatment. We observed a 70% and 30% survival rate after treating 40 μM sorafenib for 24 and 48 h in TW-1. However, the survival rate was only 28% and 3% in HTC (Figure 4A). Microarray data showed that the expression of several oncogenes (such as *c-Jun* and the *Rab* family) decreased in sorafenib-treated TW-1 compared to a no-treatment control. Moreover, many drug-resistant genes overexpressed in sorafenib-treated TW-1 (Figure 4B). These results suggested that sorafenib induced drug-resistance in TW-1 by altering its gene-expression network.

To study TW-1-associated drug resistance in vivo, we transplanted TW-1 cells into nude mice and treated them with or without sorafenib. Surprisingly, tumor sizes showed a significant dose-dependent correlation with sorafenib concentration (Figure 4C), indicating that TW-1 cells became more drug-resistant in response to a higher dosage of sorafenib. In addition, we found that TW-1-derived tumors invaded lymph nodes and metastasized to the lungs (Figure S4A,B).

### 3.4. TW-1 Cells Showed Metastases in Wild-Type Rats

**Figure 5:**
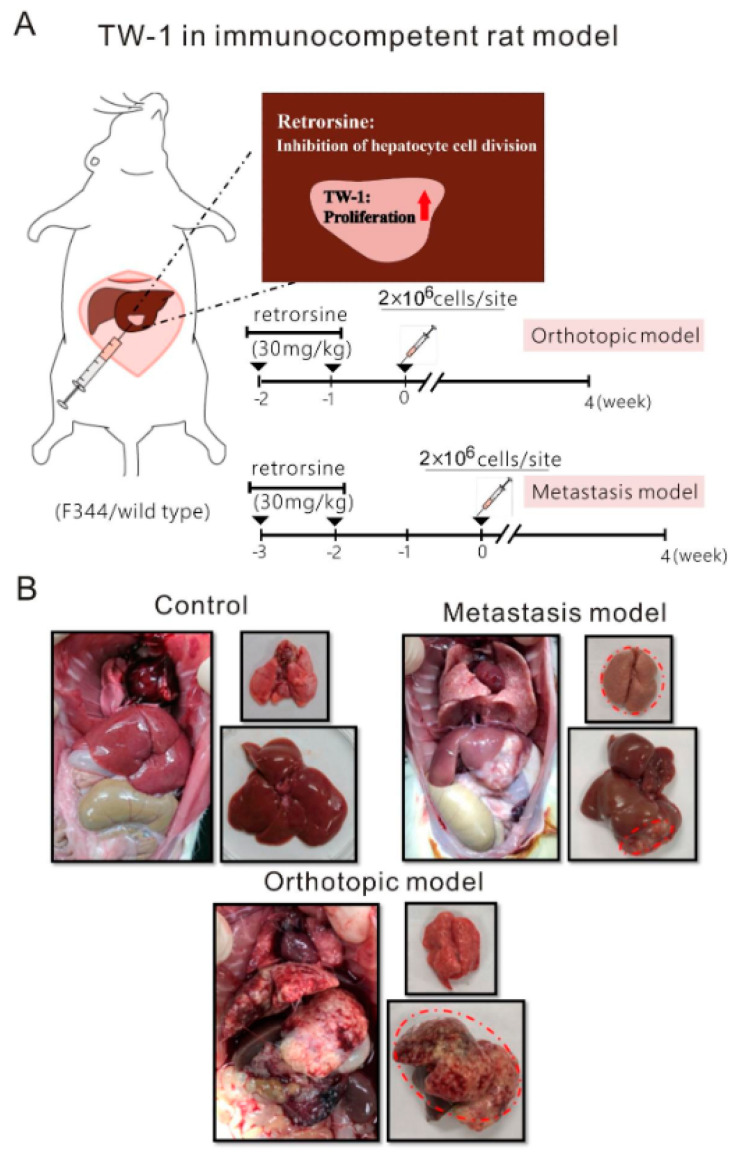
Metastasis of TW-1 in immunocompetent wild-type rats. (**A**) The schematic design of the orthotopic and metastasis rat models. TW-1 cells were injected into the liver of an immunocompetent rat one or two weeks after the retrorsine treatment. (**B**) Rats were sacrificed 4 weeks after the TW-1 injection for both models.

Our microarray data showed that genes involved in immune response, including COXII, IL33, Mal2, tetraspanin 8, and CD55, were upregulated in TW-1 compared to HTC (Table 1). We hypothesized that TW-1 cells developed into tumors by escaping from immunological detection in wild-type rats. To study the orthotopic and metastasis potential, we injected TW-1 cells into the livers of five immunocompetent wild-type rats at two different time points after retrorsine treatment. All rats developed orthotopic liver tumors or in situ liver tumors with additional lung metastases four weeks after injecting TW-1 cells, which suggested that we successfully established novel orthotopic and metastasis models in immunocompetent rats (Figure 5A,B).

### 3.5. Prediction of HCC with Machine Learning Classifiers

**Figure 6:**
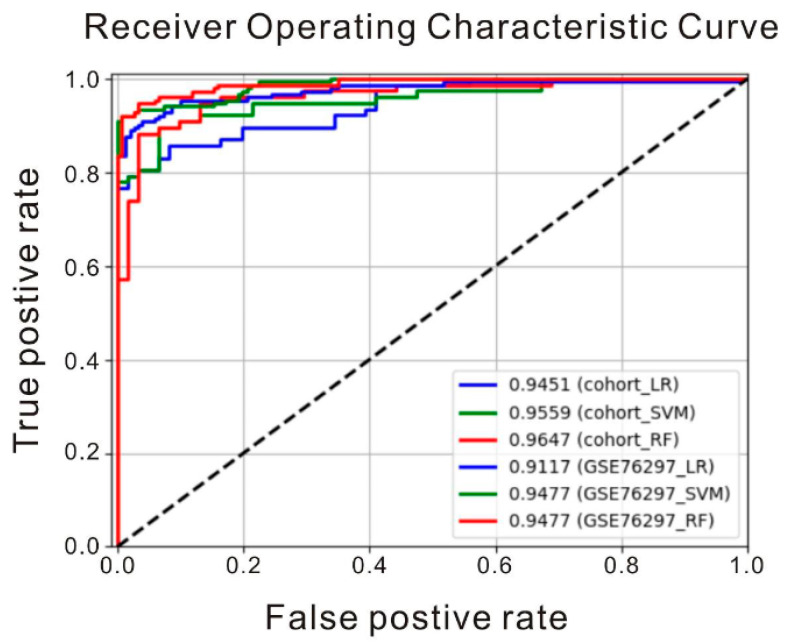
Classification of tumor types using deep learning algorithms. Dataset of an HCC human cohort was used to examine the discriminative power of the biomarkers with high singular AUC scores. Among these biomarkers, six genes (TSPAN8, RBBP5, ENO1, CD55, S100A10, and GSTA1) that showed significant differences between HTC and TW-1 microarray data and two common HCC markers (IL33 and CD44) were selected to train machine learning classifiers for HCC prediction. Three types of machine learning classifiers (logistic regression (LR), support vector machine (SVM), and random forest (RF) were used for each dataset. The predictive power of the classifiers was shown for a human HCC cohort and the human HCC microarray dataset GSE76297.

Machine learning algorithms are powerful tools that can accurately predict clinical outcomes by capturing complex interdependencies among selected biomarkers of input datasets [21]. To build a set of crucial biomarkers for machine learning, we combined two well-known biomarkers IL33 and ENO1 from recent HCC studies [22,23,24] with genes that showed high ratio of expression in our microarray data of TW-1/HTC, including TSPAN8, RBBP5, CD44, CD55, S100A10 and GSTA1, which all achieved high single AUC scores. To predict the diagnostic characteristics of HCC, we trained machine learning classifiers with an HCC cohort consisted of four open-access microarray datasets GSE6764, GSE14520, GSE60502, and GSE62232, and applied GSE76297 for independent validation. Our result showed that we were able to identify HCC with AUCs > 0.9 (Figure 6), which suggested our animal HCC model was able to apply to human HCC.

## 4. Discussion

In this study, we isolated a novel liver cancer cell line TW-1 that showed stem cell characters, including self-renewal, proliferation, cell differentiation, drug resistance, and metastasis. TW-1 expressed various markers that are unique in stem cell, such as CD133, GSTP1, CD44, and EpCAM, which was identified as a cancer cell line with progenitor or stem cell-like properties. Furthermore, we established an orthotopic and metastasis model by inoculating TW-1 cells in immunocompetent rats instead of convention model in immune deficient animals. Compared to other xenograft models, our model was benefited by an early onset of tumorigenesis, which reflected the rapidly progressing characteristics of TW-1 cancer. In addition, the benefit of using machine learning is that our predicted cancer markers can be a promising index helping oncologists and physicians develop personalized therapy and build the foundation of precision medicine for cancers that caused by cancer stem cells in the future.

### 4.1. Drug Resistance and Cancer Recurrence

It has been extensively demonstrated that therapeutic resistance accounts for the majority of ineffective clinical treatments, and the recurrence of malignant tumors poses tremendous problems for the prognosis of cancer patients. Currently, 90% of failures in chemotherapy during the invasion and metastasis stages of cancer are related to drug resistance [25]. One mechanism that triggers drug resistance is the metabolic inactivation of anticancer drugs utilizing the CYP system, GST superfamily and the uridine diphospho-glucuronosyltransferase (UGT) superfamily proteins [26]. Several anticancer therapies have taken advantage of CYP variants that are preferentially expressed in tumors, either using their ability to metabolize anticancer prodrugs or as gene therapy targets [27]. The GST superfamily is a group of detoxification enzymes whose malfunction has been implicated in the etiology of neurodegenerative disease, sclerosis, asthma, and the development of resistance to chemotherapy. Besides inactivating anticancer agents, the GSTs reduce cytotoxicity of these agents by inhibiting the JNK and p38-mediated apoptosis downstream of the mitogen-activated protein kinase (MAPK) signaling pathway [28]. Therefore, the synergistic effect of toxin detoxification and MAPK inhibition is worth considering when developing novel therapeutic agents toward the GSTs. Glucuronidation of anticancer drugs by the UGTs also contributes to drug resistance [29]. Besides drug modification and inactivation, cancer cells may acquire drug resistance by epigenetic alteration, DNA repair enhancement, or amplification of copy numbers of the oncogenes [30].

### 4.2. Liver Cancer Stem Cells and Mitochondrial Metabolism

While numerous efforts are made to combat drug resistance in the CSCs, the idea of mitochondria as therapeutic targets has recently been developed in clinical trials [31]. The CSCs are shielded in the tumor microenvironment (TME) from immune surveillance and are equipped with tools like multidrug transporters to escape from drug-elicited DNA damage [32]. One tool the CSCs rely on to facilitate tumor progression is the reprogramming of bioenergetics in response to the anaerobic TME. In addition to increased lactate production through glycolysis, the CSCs adapt to metabolic needs by altering the rate of oxidative phosphorylation (OXPHOS) in the mitochondria [33,34,35,36,37]. Drugs that suppress ATP production are well suited as potential anticancer agents. Since sorafenib enhances glycolysis, a glucose analog 2-deoxy-D-glucose that inhibits glucose uptake has been found to induce apoptosis of liver CSCs in the presence of sorafenib [38]. Therefore, a combination of chemotherapeutic agents and inhibitors of glycolysis or OXPHOS might be more effective in targeting liver CSCs [38,39].

### 4.3. Metastasis Model in Immunocompetent Rats

Malignant tumors develop metastasis when cancer cells spread from a primary site to surrounding and distant tissues. Metastasis of liver cancer cells is the main reason for poor prognosis in HCC patients. The TW-1 cell line we isolated from a DEN-induced HCC rat model was able to form malignant tumors in vivo followed by metastasis. When injected into the liver of a wild-type rat, the TW-1 cells formed tumors in the liver, lymph nodes, and lungs approximately one month after the injection. We reasoned that the TW-1 cells exhibited CSC characteristics. Although a CSC-induced cancer metastasis model has been previously reported, it was established in immunodeficient animals [40]. In contrast, our model established in wild-type rats provides an opportunity to study the mechanisms of metastasis under a physiological condition that mimics real cancer progression.

### 4.4. Biomarkers of HCC in Clinical Application

Human AFP is one of the biomarkers used in clinical HCC diagnosis. Although AFP was overexpressed in our new HTC cancer cell line, it was not expressed in TW-1. These results suggested that AFP might not be a bona fide biomarker for HCC. Recently, deep learning algorithms have achieved unprecedented success in image and speech recognition and have been applied to biological studies [41]. In this study, we successfully predicted liver cancer with AUC scores above 90% using machine learning classifiers. We could develop further applications by incorporating more biomarkers and clinical data into our deep learning models. We believe that deep learning techniques may help the physicians decide the most suitable treatments and provide a foundation of personalized therapy for individual HCC patients in the future.

## 5. Conclusions

In conclusion, our results showed that TW-1 was a novel rat CSC line, and the animal model we established with TW-1, is not only useful for the study of metastasis of HCC but also preclinical cancer drug screening.

## Figures and Tables

**Table 1:** The microarray analysis results for HTC and TW-1.

Gene ID	Fold Change (TW-1/HTC)	Gene Symbol	Description
17867292	718.25	ND2	NADH dehydrogenase subunit 2
17782314	613.15	Akr1b8	aldo-keto reductase family 1, member B8
17740581	586.22	S100a11	S100 calcium binding protein A11
17822248	573.16	Rps29	ribosomal protein S29
17880082	383.61	Rpl10	ribosomal protein L10
17867310	359.91	ND4; ND4L	NADH dehydrogenase subunit 4; NADH dehydrogenase subunit 4L
17867316	306.48	ND5	NADH dehydrogenase subunit 5
17740584	292.64	S100a10	S100 calcium binding protein A10
17863382	280.59	Gsta1	glutathione S-transferase alpha 1
17867302	252.12	COX3; ND3; ATP6	cytochrome c oxidase subunit 3; NADH dehydrogenase subunit 3; ATPase subunit 6
17867294	248.4	COX1	cytochrome c oxidase subunit 1
17846131	244.88	Gsta5; Gsta2	glutathione S-transferase alpha 5; glutathione S-transferase alpha 2
17809643	231.16	Rps8	ribosomal protein S8
17867298	229.75	COX2	COXII
17624945	165.35	Il33	interleukin 33
17829386	153.32	Mal2	mal, T-cell differentiation protein 2
17867318	150.73	CYTB	cytochrome b
17646585	146.19	Aldh3a1	aldehyde dehydrogenase 3 family, member A1
17661655	132.48	Coa3	cytochrome C oxidase assembly factor 3
17630680	131.89	Ceacam1	carcinoembryonic antigen-related cell adhesion molecule 1 (biliary glycoprotein)
17828240	125.69	Tspan8	tetraspanin 8
17683922	120.28	Cd55	CD55 molecule, decay accelerating factor for complement
17733363	118.79	Nqo1	NAD(P)H dehydrogenase, quinone 1
17832979	112.26	Rpl41	ribosomal protein L41
17715346	108.73	Serpinb6	serpin peptidase inhibitor, clade B (ovalbumin), member 6
17721215	106.48	Prtfdc1	phosphoribosyl transferase domain containing 1

## Supplementary Materials

The following are available online at https://www.mdpi.com/2072-6694/12/12/3721/s1, Figure S1: Tumorigenesis of various HCC originated from the DPPIV deficient F344 rats, The scheme of DEN, Figure S2: The original western blot pictures, Figure S3: Various cell linages were differentiated by TW 1 cells after cell culture, Figure S4: TW 1 derived tumors showed invasion in lymph nodes (A) and metastases to the lungs (B).

## Abbreviations

HCC	Hepatocellular carcinoma
CSCs	Cancer stem cells
EpCAM	epithelial cell adhesion molecule
DEN	Diethylnitrosamine
RMA	multi-array average
LR	logistic regression
SVM	support vector machine
RF	random forest
ROC	receiver operating characteristic
AUC	area under the ROC curve
AFP	alpha-fetoprotein
ABC	ATP-binding cassette
CYP	cytochrome P450 system
GST	glutathione-S-transferase
UGT	uridine diphospho-glucuronosyltransferase
MAPK	mitogen-activated protein kinase
TME	tumor microenvironment
OXPHOS	oxidative phosphorylation
