# Supplementary material for: Establishment of an Immunocompetent Metastasis Rat Model with Hepatocyte Cancer Stem Cells
Source: Cancers (Basel). 2020 Dec 11;12(12):3721. doi: 10.3390/cancers12123721 (PMC7764036; doi:10.3390/cancers12123721)
Supplement: Supplementary file 1 [file cancers-12-03721-s001.pdf]

Supplementary Materials:

Establishment of an Immunocompetent Metastasis Rat Model with Hepatocyte Cancer Stem Cells

Semon Wu [1,*], I-Chieh Tseng [1], Wen-Cheng Huang [2], Cheng-Wen Su [2], Yu-Heng Lai [3], Che Lin [4], Alan Yueh-Luen Lee [5], Chan-Yen Kuo [6], Li-Yu Su [6], Ming-Cheng Lee [6], Te-Cheng Hsu [7] and Chun-Hsien Yu [8,9,*]

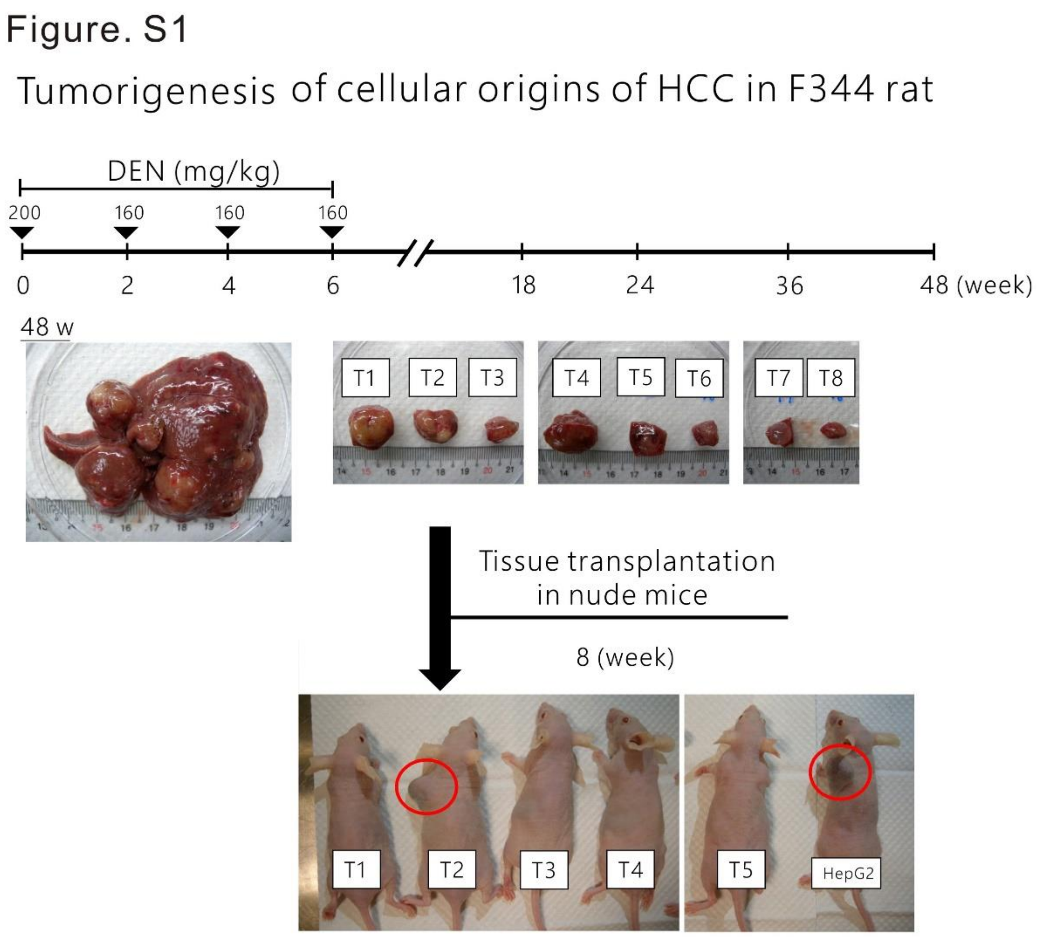

Figure S1. Tumorigenesis of various HCC originated from the DPPIV-deficient F344 rats. The scheme of DEN-induced HCC in DPPIV-deficient F334 rats was shown. The rats were sacrificed at different times after 6 weeks of DEN treatment. Tumors (T1-8) were isolated, measured, and xenografted into nude mice. Only T2 was successfully grown into a solid tumor after eight weeks. Mice injected with the liver cancer cell line HepG2 were used as control.

Figure S2.

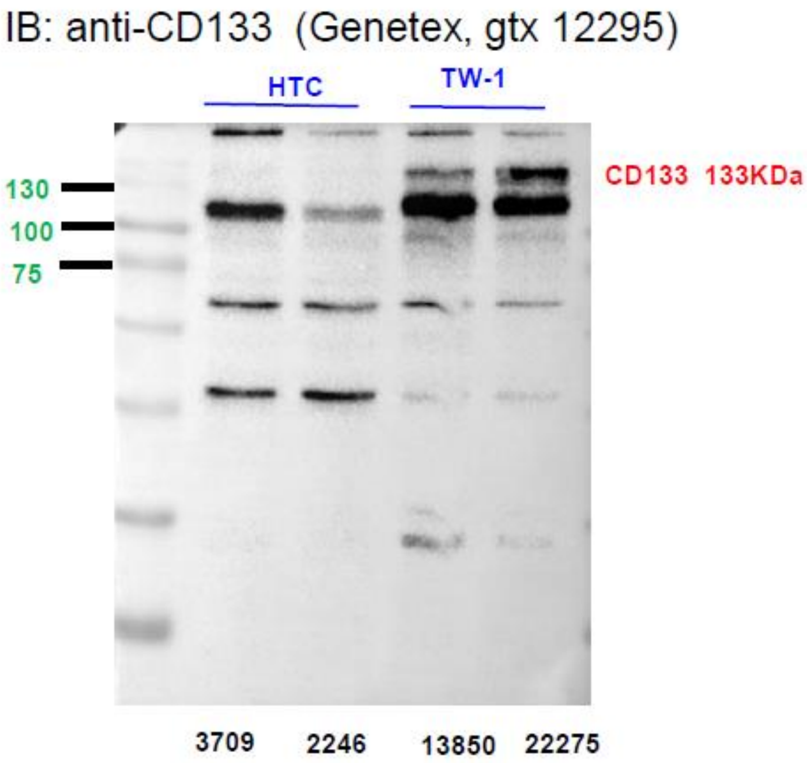

A. CD133

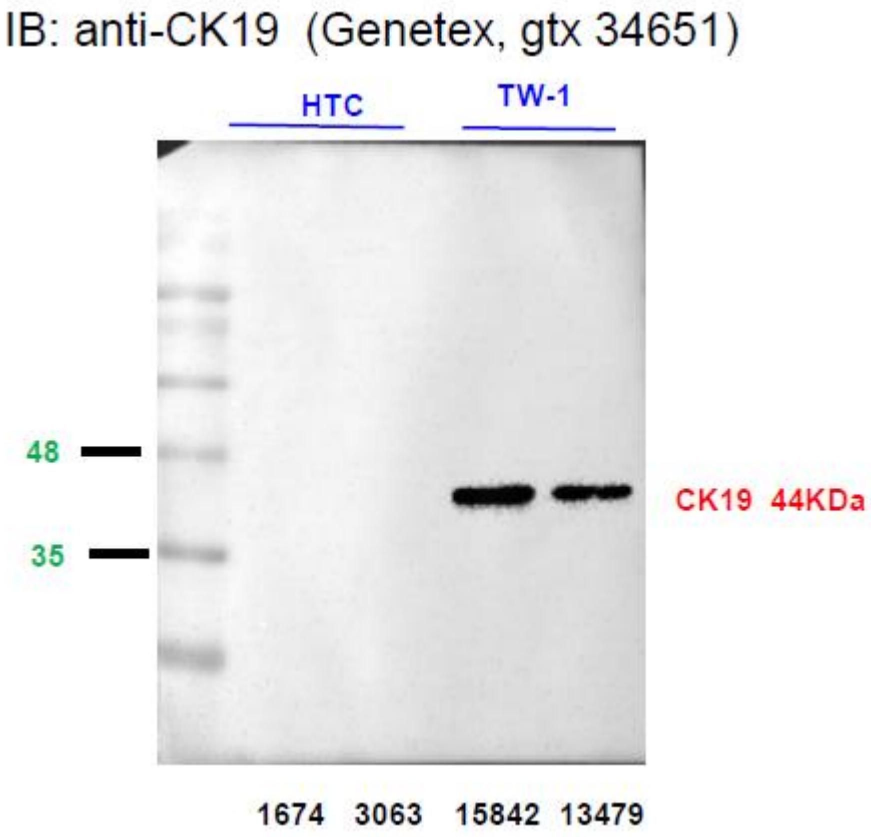

B. CK19

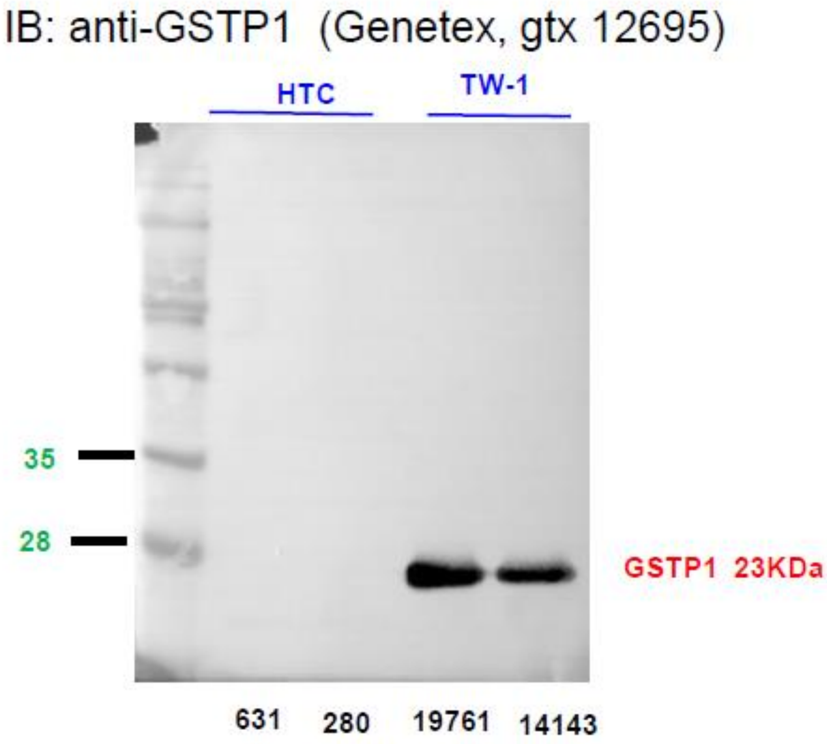

C. GSTP1

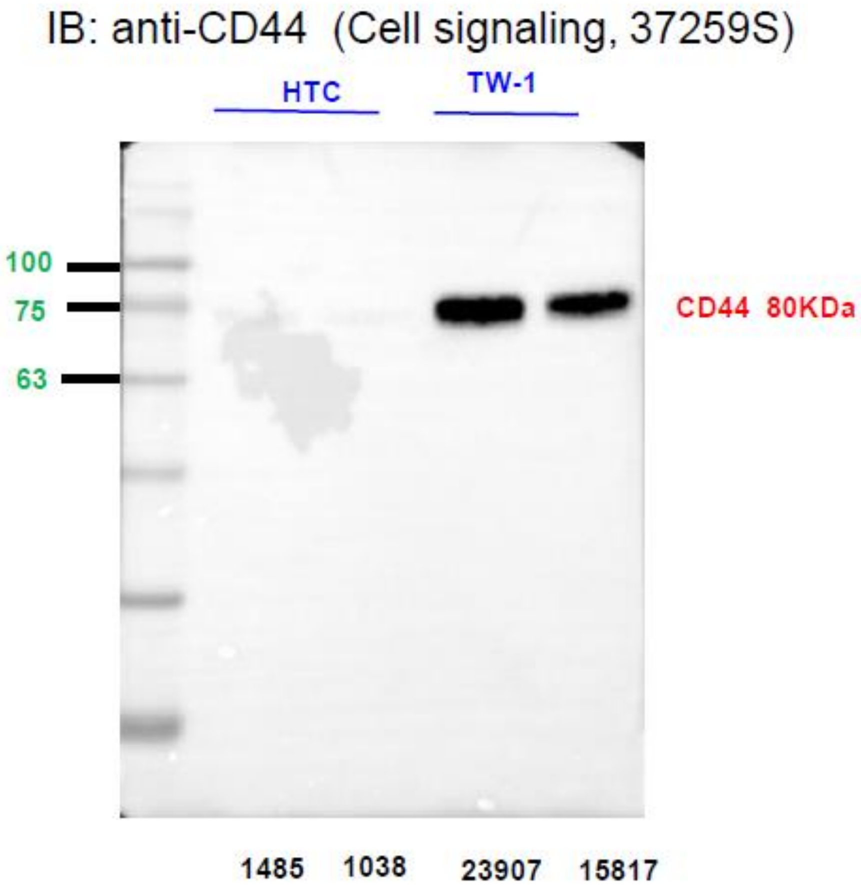

D. CD44

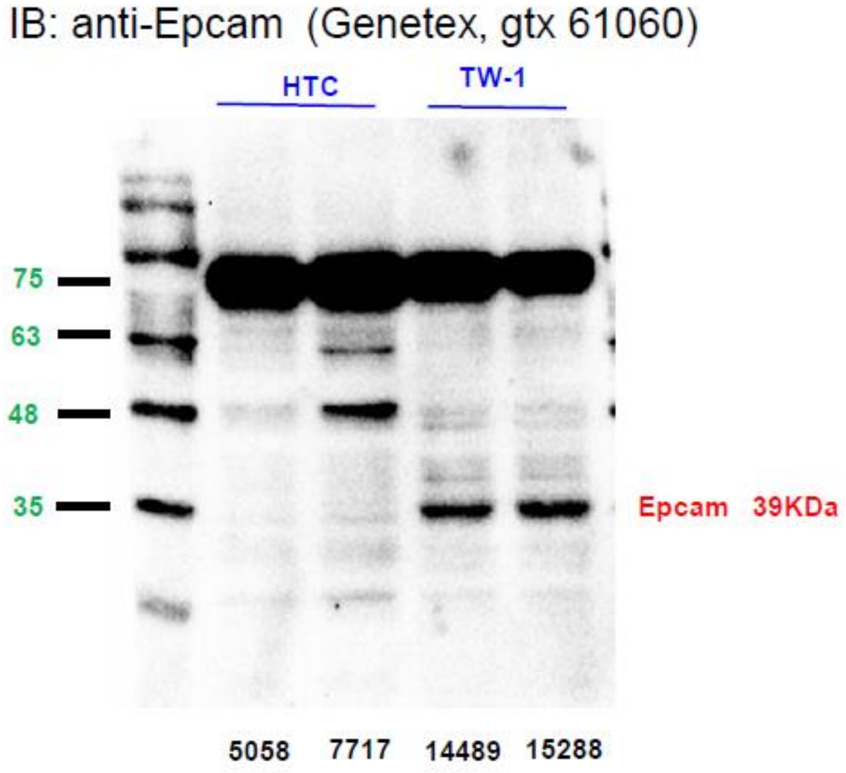

E. EpCAM

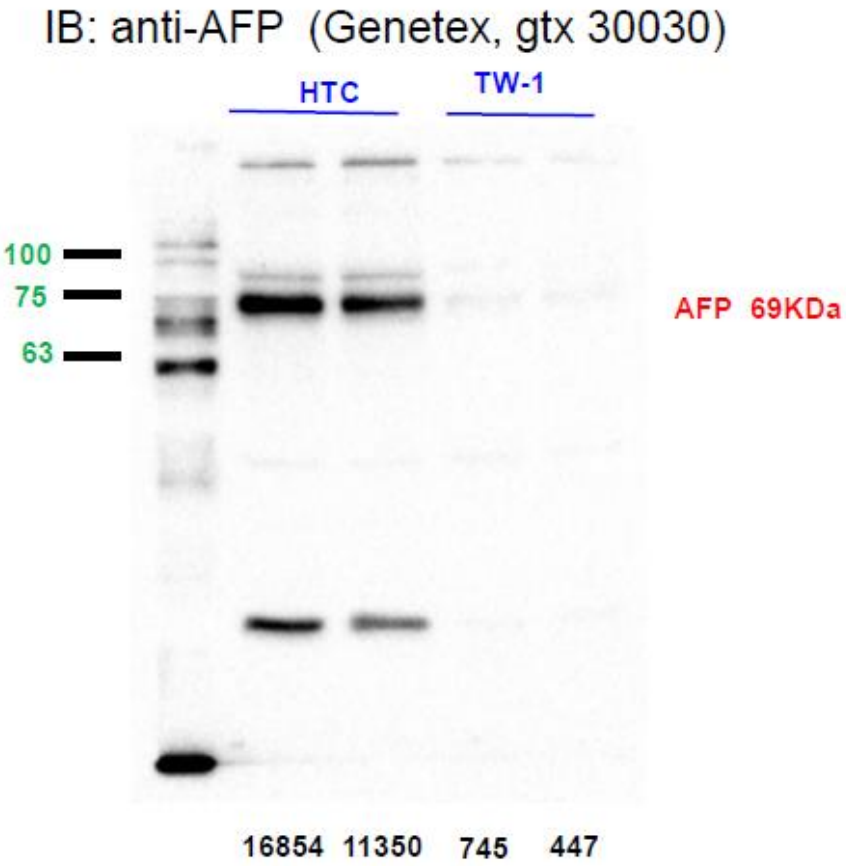
IB: anti-AFP (Genetex, gtx 30030)
HTC
TW-1
100
75
63
AFP 69KDa
16854 11350 745 447

F. AFP

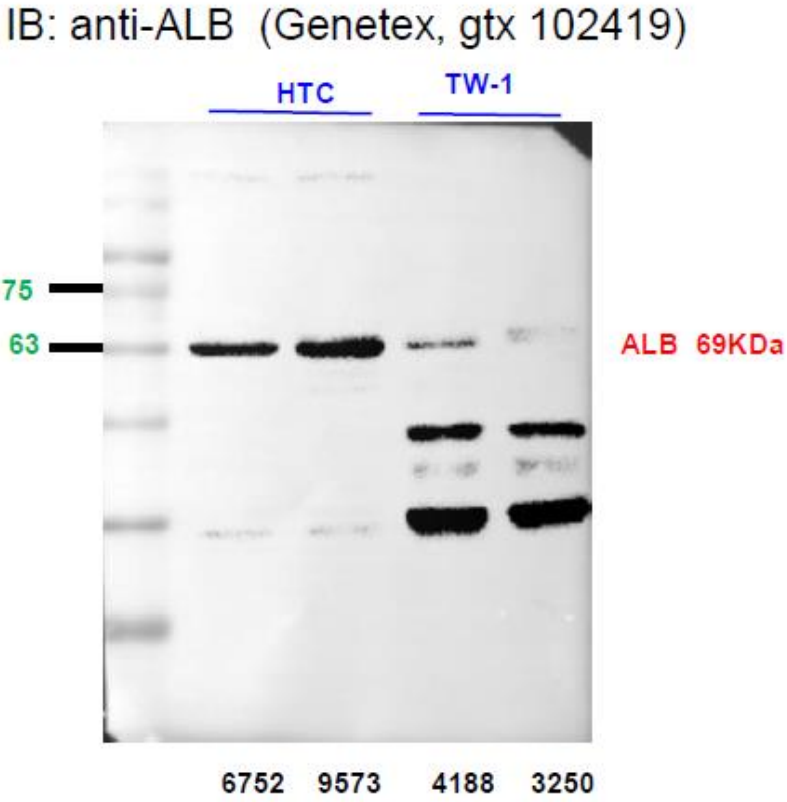
IB: anti-ALB (Genetex, gtx 102419)
HTC
TW-1
75
63
ALB 69KDa
6752 9573 4188 3250

G. ALB

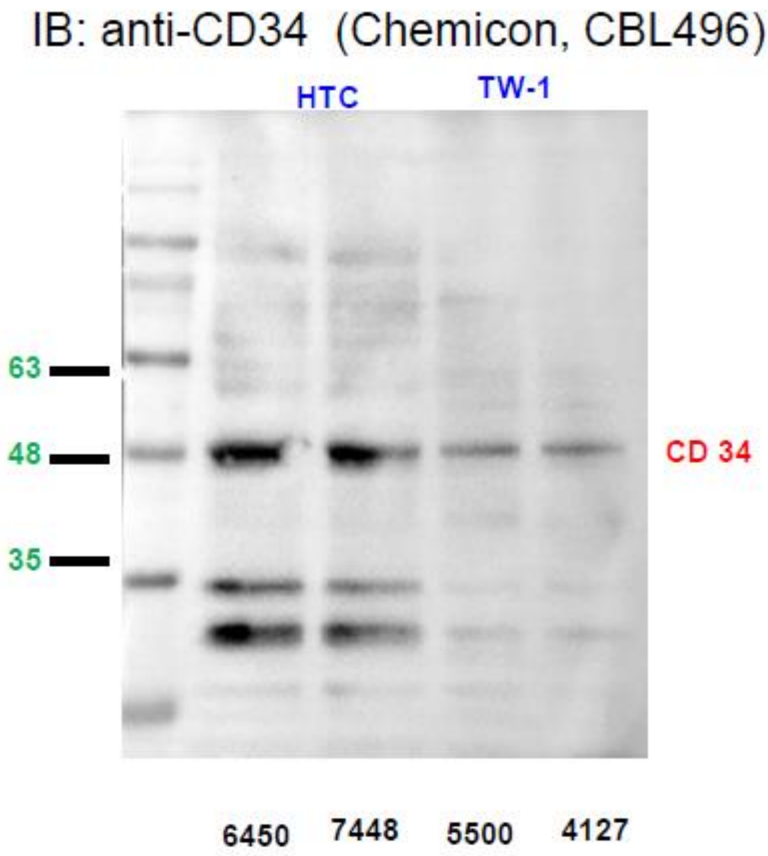

H. CD34

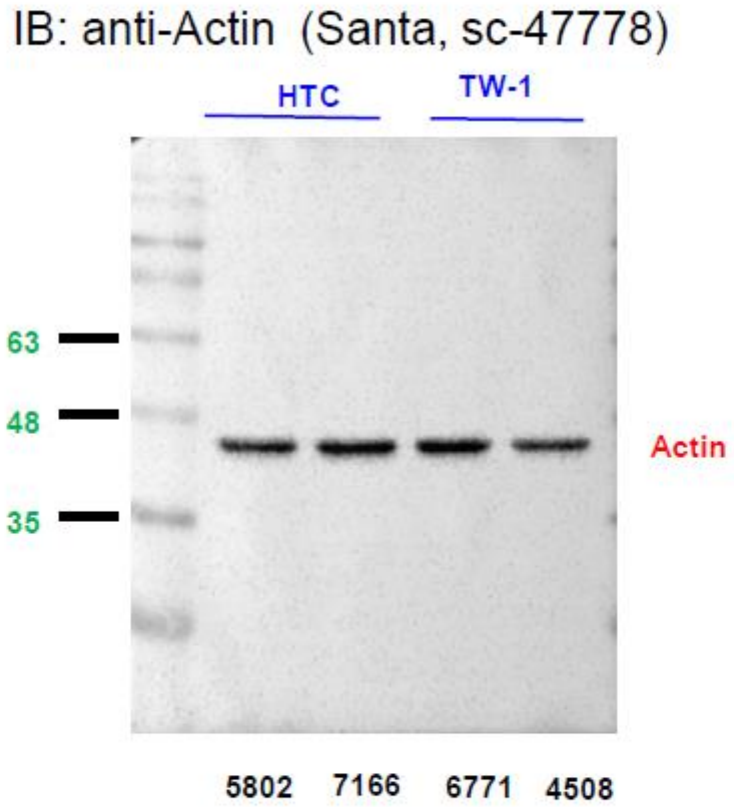

I. Actin

Figure S2. The original western blot pictures.

Figure S3.

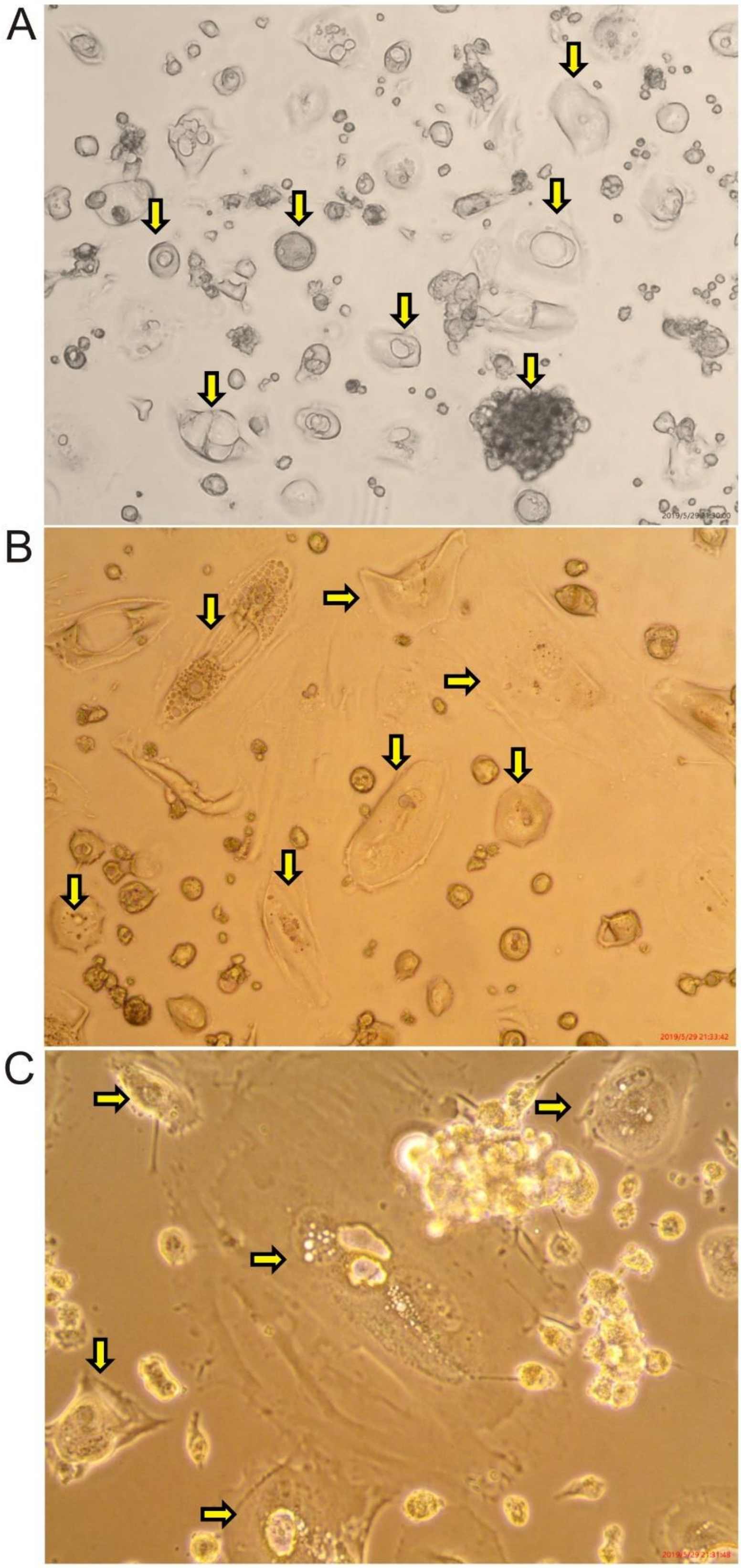

Figure S3. Various cell linages were differentiated by TW-1 cells after cell

culture.

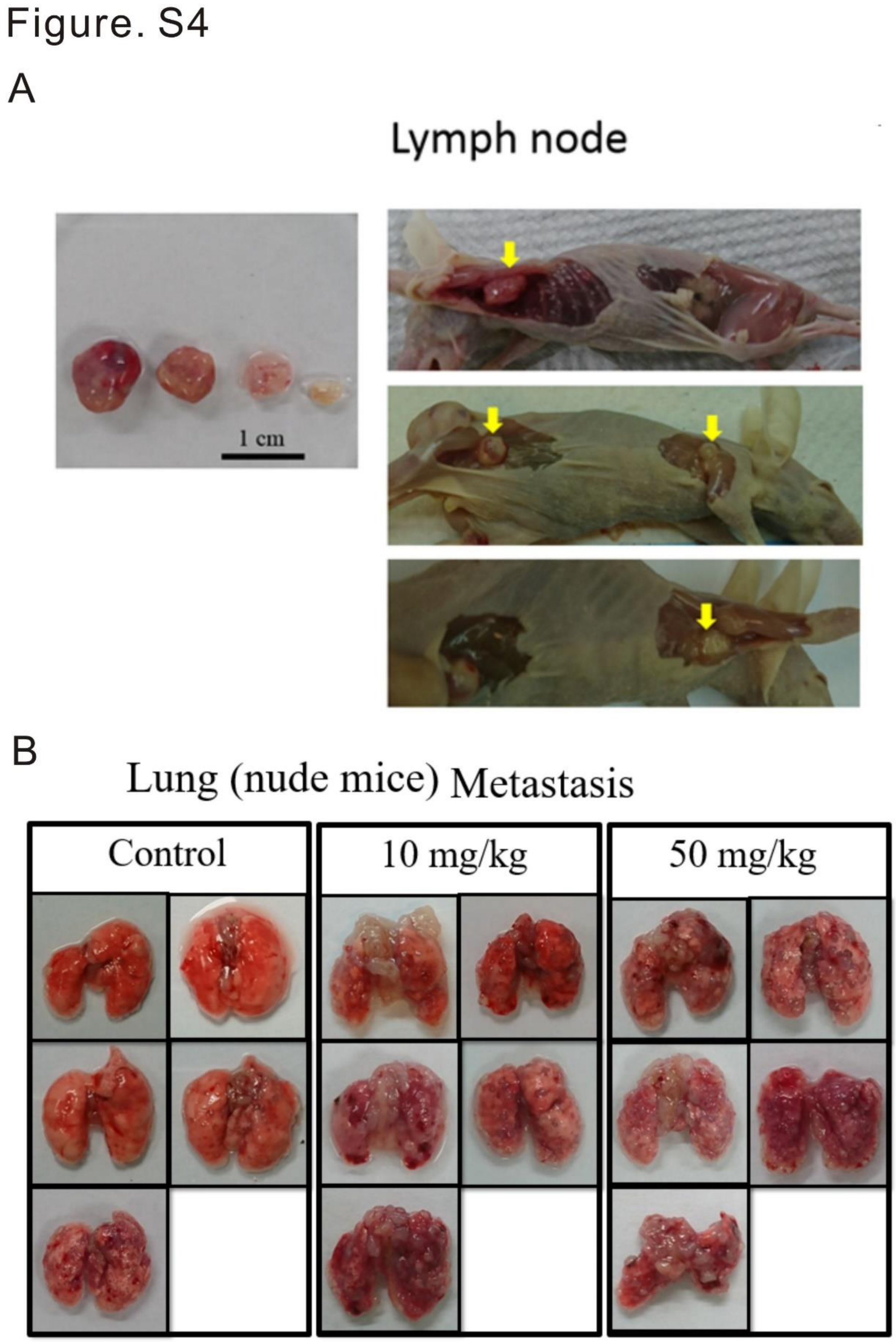

Figure S4. TW-1-derived tumors showed invasion in lymph nodes (A) and metastases to the lungs (B).
